# Modulation of Glial Cell Functions by the Gut–Brain Axis: A Role in Neurodegenerative Disorders and Pain Transmission

**DOI:** 10.3390/cells12121612

**Published:** 2023-06-13

**Authors:** Giulia Magni, Benedetta Riboldi, Stefania Ceruti

**Affiliations:** Laboratory of Pain Therapy and Neuroimmunology, Department of Pharmacological and Biomolecular Sciences, Università degli Studi di Milano, Via Balzaretti, 9, 20133 Milan, Italy; giulia.magni@unimi.it (G.M.); benedetta.riboldi@unimi.it (B.R.)

**Keywords:** astrocytes, microglia, microbiota, nociception, sensory neurons, short-chain fatty acids, dysbiosis

## Abstract

Studies on host microbiota and their interactions with the central nervous system (CNS) have grown considerably in the last decade. Indeed, it has been widely demonstrated that dysregulations of the bidirectional gut–brain crosstalk are involved in the development of several pathological conditions, including chronic pain. In addition, the activation of central and peripheral glial cells is also implicated in the pathogenesis and progression of pain and other neurodegenerative disorders. Recent preclinical findings suggest that the gut microbiota plays a pivotal role in regulating glial maturation, morphology and function, possibly through the action of different microbial metabolites, including the most studied short-chain fatty acids (SCFAs). Moreover, altered microbiota composition has been reported in CNS disorders characterized by glial cell activation. In this review, we discuss recent studies showing the role of the gut microbiota and the effects of its depletion in modulating the morphology and function of glial cells (microglia and astrocytes), and we hypothesize a possible role for glia–microbiota interactions in the development and maintenance of chronic pain.

## 1. Gut–Glia Axis in Brain Physiology and Pathology: Modulation of Glial Cell Functions by the Gut Microbiota

The human gut microbiota is a dynamic ecosystem adapted to live in the gastro-intestinal environment. It consists of different types of microorganisms, including bacteria, eukaryotes, archaea, viruses, and fungi, but it is mainly represented by bacteria belonging to 7 predominant phyla (i.e., *Firmicutes, Bacteroidetes*, *Actinobacteria*, *Fusobacteria*, *Proteobacteria*, *Verrucomicrobia*, and *Cyanobacteria*) [1]. The human microbiota is acquired during fetal life and at birth, evolves and modifies along with its host, and plays important roles in the body until adulthood and death [1].

The concept of “gut–brain axis” refers to the bidirectional communication between the gut and the central nervous system (CNS), which involves the complex integration of immunological, neural, and hormonal mechanisms and pathways [2,3]. In recent years, the dysregulation of this bidirectional communication was demonstrated to be involved in several pathological conditions, including pain, through different peripheral and central mechanisms [4]. In this respect, accumulating evidence suggests that the pivotal role played by glial cells in physiological and pathological conditions also involves their modulation by the gut microbiota, thus leading to the emerging concept of gut–glia axis whose role in various neurodegenerative disorders is currently well-established.

Conversely, a limited number of published papers on the influence of the gut–glia communication in pain transmission is currently available and, to the best of our knowledge, they have never been collected in a review article so far. Nevertheless, the pivotal entanglement of the gut–glia axis in nociception can be foreseen also based on data in neurodegenerative diseases. Thus, in this review article, we first summarize how the functions of the various glial cell populations are modified and controlled by the gut microbiota in chronic degenerative CNS pathologies. Then, in the second part of the paper, we collected the few currently available demonstrations of how the gut microbiota interacts with glial cells in painful conditions.

### 1.1. Microglia

Microglia are resident macrophage-like cells that regulate physiological functions in the brain and spinal cord [5] but are rapidly activated in response to various pathological cues [6]. The recent rise in interest in gut–brain communication has led to increased research on if and how the microbiota may influence microglial morphology and functions and, in the end, may modulate CNS diseases. Recent studies have demonstrated the role of the gut microbiota as a key regulator of microglial maturation and functions, both in normal conditions and in CNS disorders characterized by glial activation. Microglial cells are able to interact with the gut microbiota thanks to their immune nature, their sensitivity to microbial metabolites, and their primary activity of constantly monitoring the CNS [7].

To study whether the gut microbiota could influence microglia maturation and activation in mice, a transcriptomic approach showed the presence of substantial differences in the structure and function of microglia cells derived from specific pathogen-free (SPF) and germ-free (GF) mice, both at the genetic and morphological levels. Among the downregulated transcripts of GF microglia, many genes related to cell activation were identified (i.e., *Mapk8*, *Fcgr2b*, *Il1a*, *Ly86*, *Cd86*, *Hif1a*). Moreover, in these mice, the absence of a complex host microbiota increased the microglial population, caused defects in their maturation, activation, and differentiation, and altered their morphology, showing longer processes and increased branching. These alterations in microglial phenotype were reversed by recolonizing gut microbiota [8].

To assess whether gut microbiota also contributes to age-related changes in microglia physiology, a study comparing young and aged SPF and GF mice demonstrated that in aged SPF mice microglia show morphological changes (i.e., reduction in total branch area, length, and number of branch points, together with an increase in cell body volume), while microglia of aged GF mice preserved a ramified morphology. Moreover, the absence of gut microbiota reduced oxidative stress and improved mitochondrial dysfunction in aged microglial cells. These data indicate that the gut microbiota induces microglial activation and dysfunctions which are typical of aging, thus suggesting a possible role in age-related neurodegenerative disorders [9].

Since microbiota and microglia are physically and spatially separated, their reciprocal interactions are only possible through circulating factors or thanks to the activity of intermediate cells. Metabolites produced by the microbiota can indeed diffuse across the gut barrier, enter the systemic circulation and reach the CNS, where they can either act directly on microglial cells to alter their physiology or induce changes in astrocytes or immune cells to which microglia are sensitive [10]. Circulation of microbe-derived neurotransmitters, including serotonin synthesized by *Enterococcus* and *Streptococcus*, and dietary tryptophan (a serotonin precursor) metabolized by the gut microbiota, can potentially influence microglial activation either directly or indirectly [11]. A recent study in an animal model of multiple sclerosis showed that dietary tryptophan metabolites produced by the gut microbiota control microglial activation and the release of TGF-α and VEGF-β, thus attenuating the ability of microglia to induce pro-inflammatory responses in astrocytes and in the end ameliorating the disease [12].

However, the most studied mechanism regarding microbiota–microglia interaction involves circulating short-chain fatty acids (SCFAs), byproducts of bacterial dietary fiber fermentation produced by microorganisms in the intestinal lumen [10,11]. The main SCFAs are acetate, propionate, and butyrate. SCFAs can exert physiological effects in the CNS by two different mechanisms: the inhibition of histone deacetylases or the activation of a G-protein-coupled receptor named free fatty acid receptor 2 (FFAR2), primarily located in the gut [11] whose expression, however, has not yet been demonstrated on microglial cells.

In one study, a mix of the three main SCFAs was administered in drinking water to GF mice for 4 weeks. This treatment was able to reverse the deficits in microglia density, morphology, and maturity, and to rescue their immature genetic and morphological phenotype [8]. However, the exact mechanism by which SCFAs modulate microglial maturation has yet to be fully understood. In the same study, it has been demonstrated that SPF mice lacking FFAR2 exhibited a microglial phenotype similar to that observed in GF mice [8]. The absence of FFAR2 expression on microglia suggests that SCFAs may influence microglial maturation through signals that originate in the GI tract. However, how this signal propagates to the CNS and modulates microglial functions is poorly clear. It was recently discovered by the same authors that acetate alone is able to reverse many altered features of the microglial phenotype in GF mice, while propionate and butyrate are completely ineffective, indicating that acetate is an essential bacterial-derived molecule and may be the primary mediator of microglia–microbiota signaling [13]. These authors demonstrated that acetate drives microglia maturation and regulates its homeostatic metabolic state, as it can be re-uptaken within the microglial Krebs cycle and used in the TCA cycle [13]. Nevertheless, the mechanism by which acetate affects microglial phenotype remains largely unclarified though several potential pathways have been hypothesized. Another paper evaluated the anti-inflammatory effects of SCFAs on LPS-stimulated microglia in mice that consumed inulin, a soluble fiber that is fermented by the gut microbiota to produce SCFAs. Microglia isolated from these mice and stimulated with LPS in vitro showed a lower secretion of TNF-α. Similarly, in vitro treatment of primary microglia with acetate and butyrate, alone or in combination, reduced microglia cytokine production, histone deacetylase activity, and NF-kB nuclear translocation, demonstrating that a diet that increases SCFAs inhibits microglia inflammation. Once again, the expression of FFAR2 was not detected on microglia, differently from SCFA transporters Mct1 and Mct4. However, their inhibition did not interfere with the anti-inflammatory effects of SCFAs, suggesting that they probably regulate microglia functions through an epigenetic mechanism following transmembrane diffusion [14].

Microglia cells have been extensively studied in the context of CNS diseases, given their vital role in maintaining CNS homeostasis, which is impaired under pathological conditions. In fact, microglia activation is involved in the initiation or progression of several CNS neurodegenerative and neuroinflammatory diseases such as Alzheimer’s and Parkinson’s diseases (AD; PD) [7].

Several pieces of evidence also correlate alterations in the gut microbiota with the progression of neurodegenerative diseases [15]; in fact, microbiota activates the immune system as a consequence of alterations in the gut barrier, leading to systemic inflammation that causes damage to the blood-brain barrier (BBB) and promotes neuroinflammation and neuronal degeneration. In this respect, Braniste and colleagues have demonstrated that the influence of the gut microbiota on BBB starts during prenatal life, since germ-free mice display increased BBB permeability associated with reduced expression of the tight junction proteins occludin and claudin-5 compared to mice with normal gut flora, starting from intrauterine life until adulthood. Exposure of GF adult mice to a pathogen-free gut microbiota decreased BBB permeability and up-regulated the expression of tight junction proteins, further demonstrating the positive effect of the microbiota on BBB functions [16]. As described above, microbiota-derived metabolites have also been shown to regulate the inflammatory response mediated by microglial cells, thus suggesting that microglia may act as a critical mediator between the gut and the CNS under pathological conditions.

We first analyze a few papers regarding microglia–microbiota interactions in AD pathology, followed by a review of some results about these interactions in PD.

A study conducted in a recently developed ADLP^APT^ (AD-like pathology with amyloid and neurofibrillary tangles) transgenic mouse model of AD showed the presence of amyloid-β plaques, neurofibrillary tangles, and reactive gliosis in the brain, along with a different gut microbiota composition with respect to healthy wild-type (WT) mice. Fecal microbiota transplantation (FMT) from WT to transgenic mice resulted in a lower aggregation of amyloid-β plaques in the frontal cortices and hippocampi and reduced activation of microglia and astrocytes [17].

In a second study that employed APP/PS1 transgenic mice, the most common animal model of AD, the intragastrical treatment with *Clostridium butyricum* (CB) prevented cognitive impairment, amyloid-β deposits, microglia activation, and the brain production of TNF-α and IL-1β. In addition, abnormal gut microbiota and abnormal levels of butyrate were reversed by CB treatment. In the same study, pretreatment of amyloid-β-activated microglia with butyrate reduced CD11b and COX-2 levels and inhibited the phosphorylation of the p65 subunit of NF-kB, suggesting that the effects of butyrate against AD neuroinflammation could occur through suppression of NF-kB signaling [18]. These results indicate that CB treatment attenuates microglia-mediated neuroinflammation by regulating the gut–brain axis through the metabolite butyrate.

Another study conducted in the same animal model of AD showed that GF AD mice exhibit a substantial reduction in amyloid-β deposition and SCFA plasma concentration. Conversely, supplementation with SCFAs for 8 weeks increased cerebral amyloid-β loads and resulted in specific activation of microglia associated with an up-regulation of the ApoE-Trem2 signaling in the brain, thus mimicking the effect of microbiota on these cells. However, despite increased microglial recruitment to amyloid-β plaques upon SCFA supplementation, it has been also demonstrated that microglia contain less intracellular amyloid-β. Taken together, these results suggest that microbiota-derived SCFAs are critical mediators along the gut–brain axis, promoting amyloid-β deposition likely through the modulation of the microglial phenotype [19].

Another research group studied the effects of the constitutive or induced modulation of the microbiota in the 5 × familial AD (5 × FAD) transgenic mice. They showed that both GF 5 × FAD mice and antibiotic-treated 5 × FAD mice showed attenuation of amyloid-β accumulation and associated neuronal loss in the hippocampus, thereby delaying disease-related memory deficits. However, only in GF 5 × FAD mice, an increase in the uptake of amyloid-β by microglia in the early stages of the disease has been observed. Indeed, RNA sequencing of microglia in the hippocampus of these mice revealed increased expression of phagocytosis-related genes. Thus, it can be hypothesized that the gut-derived amelioration of AD pathology in GF mice results from the regulation of amyloid-β clearance from microglial cells. These results suggest that constitutive or induced microbiota depletion differentially controls microglial amyloid-β clearance mechanisms, preventing neurodegeneration and cognitive deficits [20]. The same authors also studied the effects of oral administration of acetate, which was found able to modify amyloid-β accumulation, reduce microglial phagocytosis in GF 5 × FAD animals, and modulate the progression of AD by shaping microglial innate immune mechanisms. In fact, acetate induces microglial pro-inflammatory phenotype with increased cytokine expression, which, in turn, attenuates microglial phagocytosis [13]. These results appear to be in contrast with data reported by Sun and colleagues (see above [18]); however, authors speculated that this discrepancy could be due to the different AD models employed in the two studies, as well as to disease stage-dependent divergencies [20].

Regarding studies on the role of microglia–microbiota interactions in PD, it has been demonstrated that FMT from healthy mice to mice injected with 1-methyl-4-phenyl-1,2,3,6-tetrahydropyridine (MPTP), a model of toxin-induced PD, attenuates motor deficits, glial activation in the *substantia nigra*, and reduces Toll-like receptor 4 (TLR4)/TNF-α signaling pathway in the gut and brain. Furthermore, this study showed that higher concentrations of fecal SCFAs in mice with PD correlate with higher levels of activated striatal microglia and astrocytes, thus demonstrating that fecal SCFAs may contribute to the over-activation of glial cells in the *substantia nigra*, while FMT may exert a neuroprotective effect thanks to a normalization of the whole signaling pathway [21].

Finally, one group demonstrated that GF transgenic mice overexpressing human α-synuclein showed a reduction in motor deficits, α-synuclein inclusions, and microglial activation compared with mice with complex microbiota. However, the administration of a mix of three SCFAs to these mice promoted neuroinflammation, by increasing microglial activation, and induced PD-related motor symptoms similarly to animals that did not undergo microbiota depletion [22]. These data suggest that microbiota is essential for PD pathogenesis and that its metabolites could drive pathological microglial activation.

Interestingly, when obese-insulin-resistant male rats were treated with the prebiotics Xylooligosaccharide, or the probiotic *Lactobacillus paracasei* HII01, or a combination of the two (a so-called synbiotic combination) a reduction in hippocampal oxidative stress, apoptosis, and microglia activation was observed, leading to the restoration of cognitive function thanks to the interaction between microbiota and microglial cells across the gut–brain axis [23].

Overall, these data support the hypothesis that the microbiota may drive the development of neurodegenerative diseases through multiple mechanisms, including microglial cell activation mediated by host microbiota metabolites.

### 1.2. Astrocytes

Astrocytes are the most abundant glial cell population in the CNS and play a vital role in maintaining CNS homeostasis. After any traumatic or ischemic damage, astrocyte phenotypes and functions can undergo significant changes, overall known as reactive astrogliosis which limits the spreading of damage and represents a first attempt towards regeneration, but can also contribute to cytotoxicity [24].

Although the supporting literature is not as extensive as in the case of microglia, several papers have recently shown that the gut–brain axis can modulate astrocyte maturation and functions as well, via multiple mechanisms which include activation of immune pathways, the release of cytokines and inflammatory mediators, activation of TLRs, modulation of the release of neurotransmitters and neuroactive hormones, and the regulation of BBB permeability (for review, see [25]).

Starting from the demonstration that early dietary administration of lactic acid, a product of *Lactobacilli*, positively influences astrocyte development in rats [26], evidence showed that microbiota-released mediators are able to modulate astrocyte number, morphology and functions. Additionally, astrocyte abnormalities have been observed in disorders characterized by gut dysbiosis [25]. For example, authors observed an increase in the number of S100β-immunopositive astrocytes in the brain of children with autism spectrum disorder in parallel with gut dysbiosis [27]. In a rat model of depression-like behavior, namely the chronic unpredictable mild stress model, astrocyte activation in the hippocampus was accompanied by alterations in the composition of the gut microbiota [28]. Similarly, in male rats subjected to finasteride-induced depressive-like behavior an increased number of GFAP-immunoreactive astrocytes in the hippocampal dentate gyrus was accompanied by gut dysbiosis [29]. Moreover, an altered composition of the gut microbiota was observed in mouse models of traumatic brain injury [30], AD [31], and PD [21], also characterized by the presence of reactive astrogliosis. Recently, a research group demonstrated that a “gut microbiota-astrocyte” axis mediates neurocognitive dysfunctions secondary to metabolic disorders in diabetes mellitus [32].

Interestingly, a recent paper showed that a subpopulation of LAMP1+ TRAIL+ astrocytes limits CNS inflammation by inducing T cell apoptosis and that this astrocyte subset is maintained by meningeal IFNγ+ NK cells that are licensed by the gut microbiota [33].

As a further confirmation of the regulation of astrocyte maturation and function by the gut microbiota, supplementation of the diet with a bioactive food reduced astrocytic/microglial activation in the brain of AD mice, thus improving amyloid-β aggregation and tau hyperphosphorylation [34]. Moreover, female mice fed with high fat and high sugar diet displayed a reduced astrocytic density in the hypothalamus, paralleled by an increased abundance of *Bacteroidetes* and *Firmicutes* in the gut microbiota [35].

Noteworthy, astrocyte activation is also regulated by some gut microbiota-derived metabolites, including SCFAs. For example, sodium butyrate significantly increased the number of activated astrocytes in the in vivo model of MPTP-induced PD [36]. Moreover, evidence shows that butyrate influences astrocytic functions also by regulating mitochondria activity [37]. Intracerebroventricular administration of propionic acid, another metabolic product of gut bacteria, caused neuroinflammation and reactive astrogliosis in the hippocampus and white matter in a rat model of autism spectrum disorder [38]. More recently, it was shown that a single intraperitoneal injection of propionic acid to naïve rats activates astrocytes in the amygdala [39].

Evidence also demonstrates that recovery from gut dysbiosis via different strategies, including probiotics administration and FMT, is able to reverse the pathological activation of astrocytes [25,32].

**Figure 1:**
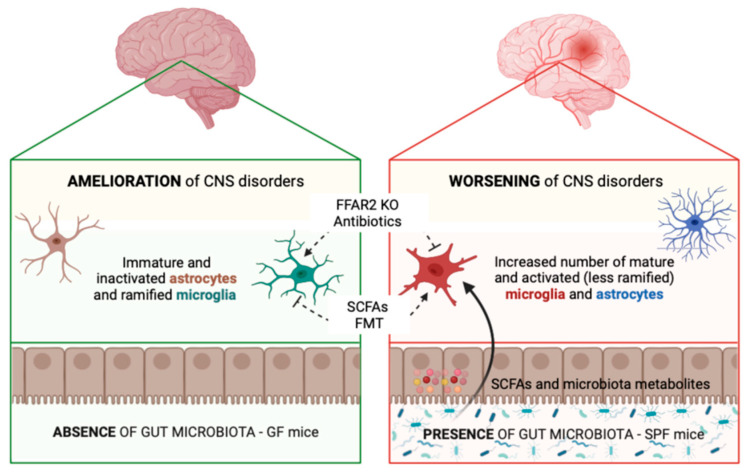
Gut microbiota modulates glial cell functions and morphology contributing to CNS disorders. Microglia and astrocytes from GF mice (**left**) show an immature and inactivated phenotype, express several immaturity markers, and elicit a dampened response to immune stimulation, while glial cells from SPF mice (**right**) display a mature and activated phenotype. Evidence shows that treating GF mice with SCFAs or recolonizing the gut microbiota via FMT can restore glial cells to a mature phenotype. On the other hand, FFAR2 KO and antibiotics-treated mice show a GF-like immature glia phenotype. In addition, consistent with results showing gut dysbiosis in diseased mice, GF mice exhibit milder CNS disorders, which are instead worsened in SPF mice. Created with BioRender.com, accessed on 28 April 2023.

Similarly to microglia, published literature suggests that regulation of reactive astrogliosis could be one of the mechanisms through which the gut microbiota participates in the development of neuroinflammation and neurodegeneration, as summarized in Figure 1. However, further studies are needed to better investigate the interactions between glial cell activation and microbiota in CNS diseases.

### 1.3. Other Glial Cells

At variance from microglia and astrocytes, literature data on the modulation of oligodendrocytes by the gut microbiota are quite scarce. Oligodendrocytes are the myelin-generating cells in the CNS with the primary functions to provide isolation and metabolic support to axons and to favor jumping transmission of electrical signals [40]. Available data point towards a role for the gut–brain axis in the maturation of this cell population and, consequently, in myelination. Indeed, experimental evidence clearly indicates that dysbiosis occurs in several disorders characterized by myelin damage or disruption. Since shaping of the gut microbiota coincides with the critical period of developmental myelination, authors speculated on the regulation of the oligodendrocyte progenitor niche by the gut microbiota via different mechanisms (for review see [41,42]). Moreover, the absence of gut microbiota in GF mice reduced callosal myelination and white matter plasticity in the prefrontal cortex, due to reduced gene expression of myelin-related proteins and reduced levels of mature oligodendrocytes [43].

If in the case of the CNS, the existence of a gut–glia axis is widely supported by experimental evidence, no literature data clearly address the role of the gut microbiota in the regulation of peripheral glial cells in health and disease. Recently, a review article collected evidence from preclinical studies showing the beneficial effects of nutraceuticals on peripheral gliopathies and associated pathological conditions, with a focus on the modulation of satellite glial cells (SGCs; see below). Although not directly addressed by literature, authors speculate a role for the gut microbiota as one of the mechanisms involved in the modulatory role of nutraceuticals on SGCs activity [44]. Besides this, preclinical data demonstrating the modulation of SGC and Schwann cell maturation and function by the gut microbiota are lacking, thus leaving an open question on the existence of a gut–glia axis in the peripheral nervous system (PNS).

## 2. Glial Cells as Key Actors in Pain Pathways

**Figure 2:**
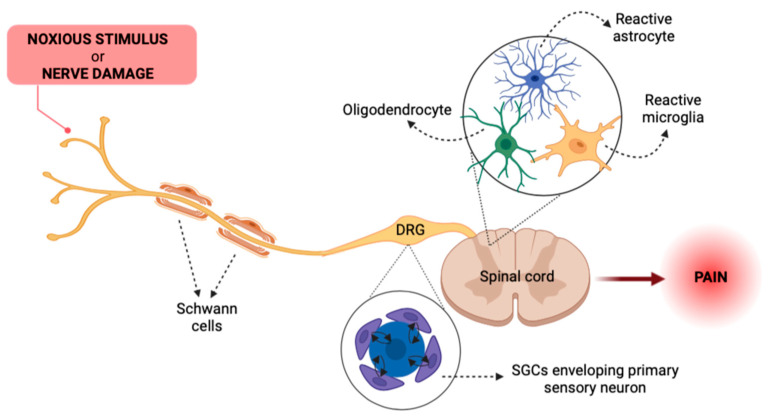
Schematic representation of glial cell populations involved in pain transmission. After painful stimuli, Schwann cells and SGCs in the PNS become activated and release pro-inflammatory mediators that sensitize nociceptors. In the CNS, astrocytes, and microglia respond to noxious stimulation changing their morphology into an activated phenotype and releasing bioactive factors. See text for details. Created with BioRender.com, accessed on 28 April 2023.

Over the past decades, it has been understood that the mechanisms underlying pain encompass neuronal plasticity phenomena in both the PNS and the CNS. Initially, synaptic changes leading to central sensitization were thought to involve only peripheral and central nociceptive neurons [45]. However, in addition to the key role of neurons, numerous studies have also demonstrated the importance of activation of non-neuronal cells, including immune (i.e., macrophages and lymphocytes) and glial cells, along pain circuits (see Figure 2), leading to neuroinflammation, a localized form of inflammation in the PNS and CNS [46]. Activation of these cell populations, in turn, leads to the generation of bidirectional interactions with nociceptors, and it is now clear how this phenomenon plays a key role in the transition from acute to chronic pain and in its subsequent maintenance [47].

In this scenario, the main non-neuronal actors in the CNS are microglia cells and astrocytes. In general, a time-dependent and coordinated sequence of events occurs; first, microglia detect painful stimuli from the periphery and respond by changing their morphology and releasing bioactive factors, which, in addition to the sensitization of second-order neurons, also promote reactive astrogliosis. Then, reactive astrocytes, in turn, further support microglia reactivity and modulate neuronal excitability leading to a complex crosstalk that promotes the development of pain and its chronicization [48,49,50].

As for other types of glial cells, the role of oligodendrocytes in pain transmission is still elusive, although their selective ablation has been demonstrated to induce pain independently from immune cell involvement [51]. This observation highlights the need for additional studies to further understand how this cell population integrates with the complex signaling pathways which control pain chronicization.

In the PNS, the main types of glial cells are Schwann cells, which provide myelin sheaths to peripheral nerves [52], and SGCs, which enwrap the cell bodies of primary sensory neurons within dorsal root ganglia (DRGs) and trigeminal ganglia (TGs) [53]. In response to painful stimuli, these cells become activated before the central glia and release inflammatory mediators that sensitize nociceptors at axons and cell bodies, contributing to both peripheral and central sensitization [47,52].

Overall, the existence of a bidirectional neuron-glia signaling both in the CNS and PNS, which is responsible for the initiation and maintenance of chronic pain conditions, has now been widely established [54].

## 3. The Gut–Brain Axis in Pain Transmission

Several studies demonstrate the key role of the gut microbiota in visceral pain: inflammatory bowel disease (IBD) patients show both gut barrier dysfunction and an altered microbiota composition (dysbiosis) compared to healthy subjects [55], and rats exposed to antibiotics early in life show increased visceral pain through alteration of gut microbiota, with an effect dependent on the time of exposure [56]. Moreover, recently published clinical studies demonstrate that targeting the gut microbiota may be a strategy for the management of visceral pain related to gastrointestinal disorders, stress response, and altered pain sensitivity [4]. Interestingly, recent discoveries regarding the human gut microbiota and visceral pain led to a hypothesis about the existence of a correlation between the gut microbiota and chronic pelvic pain, including pain related to endometriosis, via multiple mechanisms involving inflammatory response [57].

Gut dysbiosis is also related to the development and maintenance of inflammatory pain, as first demonstrated by the reduction of inflammatory hypernociception induced by carrageenan, lipopolysaccharide (LPS), TNF-α, IL-1β, and chemokine CXCL1 in GF mice [58]. In line with this, a number of studies suggest that targeting the gut microbiota with a dietary approach or probiotic supplementation can reduce pain hypersensitivity in different inflammatory settings [2]. As an example, a recent study shows that the oral administration of *Lactobacillus rhamnosus* ameliorates the progression of osteoarthritis in rats, by inhibiting joint pain and inflammation [59].

Other studies also reported the involvement of the gut microbiota in chemotherapy-induced neuropathic pain: GF mice and mice pre-treated with antibiotics displayed reduced oxaliplatin-induced mechanical hyperalgesia [60], while paclitaxel-induced neuropathic pain was counteracted by the probiotic DSF (De Simone Formulation, also known as VSL#3^®^), a high concentration probiotic formulation [61]. A recent study also showed that an altered composition of the gut microbiota contributes to neuropathic pain and anhedonia susceptibility induced by spared nerve injury (SNI) in rats [62]. By contrast, another study showed no significant effect of oral probiotics administration, such as *L. reuteri LR06* or *Bifidobacterium BL5b*, on chronic-constriction injury (CCI)-induced neuropathic pain in rats [63]. However, in a model of neuropathic pain induced by CCI of the sciatic nerve in mice, authors reported that the gut microbiota mediates pain development via modulation of pro- and anti-inflammatory T-cell responses, and the administration of oral antibiotics reduced CCI-induced neuropathic pain [64]. These apparently contradictory results suggest that interspecies differences may occur. The protective effect of probiotic supplementation has been more recently shown in both male and female mice exposed to spinal nerve transection (SNT) leading to neuropathic pain. Marked dysbiosis was in fact detected after SNT whereas mice treated with a cocktail of antibiotics were resistant to SNT-induced pain [65].

Interestingly, alterations in the composition of the gut microbiota seem to be involved also in migraine pain via multiple mechanisms [66]. In this respect, supplementation of probiotics was shown to improve the quality-of-life scores in migraine patients [67], and patients with frequent migraine have more complaints about gastrointestinal symptoms than healthy controls [68]. Moreover, a more recent study showed that the therapeutic effect of daily probiotics administration for 12 weeks in migraine patients is comparable to the effect of antiepileptic and antihypertensive drugs [69].

**Figure 3:**
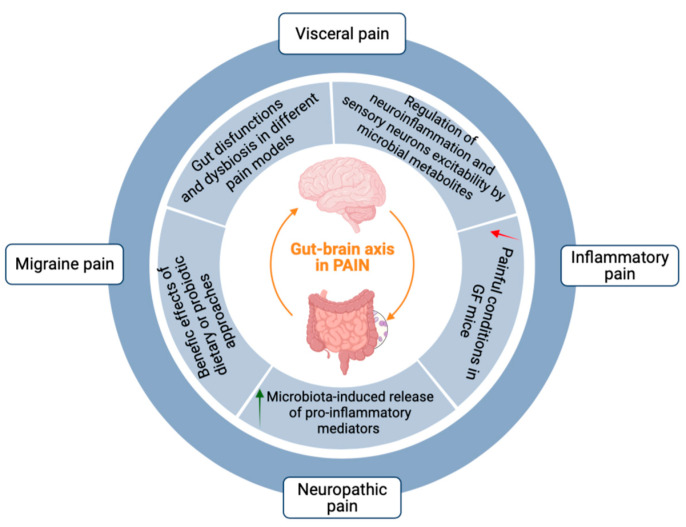
Mechanisms and experimental evidence at the basis of gut–brain communications in pain. An imbalance in the gut microbiota composition with alterations of the bidirectional crosstalk between the gut and the brain is involved in the pathogenesis and maintenance of different types of pain. Green arrow means increase, while red arrow means decrease. See text for details. Created with BioRender.com, accessed on 28 April 2023.

Taken together, evidence from recent literature suggests that restoring the composition of the gut microbiota represents an effective therapeutic approach to treating different pain conditions, thus highlighting the fundamental role of the gut–brain axis in nociception. The main mechanisms underlying the involvement of gut-brain axis in different pain syndromes are summarized in Figure 3.

## 4. Emerging Evidence for a Role of the Gut–Glia Axis in Pain Transmission

Based on the growing literature data demonstrating that the gut microbiota modulates glial cell maturation and functions and given the fact that glial cells are a key factor in pain transmission, it seems obvious to speculate on a role for the gut–glia axis in pain onset and development.

Nevertheless, very few papers directly addressed this issue so far. In a model of complete Freund’s adjuvant (CFA)-induced temporomandibular joint inflammatory pain, systemic administration of the natural bioactive compound resveratrol reduced inflammation and orofacial pain, restored CFA-caused reduction of SCFAs and recovered gut dysbiosis. Moreover, resveratrol returned CFA-enhanced microglial activation to baseline levels, and FMT from resveratrol-treated mice to CFA-injected animals attenuated TMJ inflammatory pain and inhibited microglial activation, suggesting that the beneficial effect of the compound on microglia activation occurs via the gut–brain axis [70]. Similarly, a study demonstrated that treatment with berberine reduces visceral hypersensitivity in rats by altering the composition of the gut microbiota and suppressing spinal microglial activation. In parallel to in vivo experiments, authors performed in vitro studies providing evidence that berberine inhibits microglia activation through the gut–brain axis, and not via direct effects [71].

Another paper demonstrated that SCFAs play a key role in the pathogenesis of CCI-induced neuropathic pain by regulating microglia activation and polarization towards a pro-inflammatory phenotype, and antibiotic administration reverses these alterations [72]. Conversely, despite the demonstration of a key role for gut dysbiosis in the development of neuropathic pain after SNT (see above), no modifications in microglia and astrocyte activation were observed after administration of probiotics which, however, reduced TNF-α production in the spinal cord and DRGs [65]. This apparent contradiction clearly points to a possible differential cross-talk between gut microbiota and glial cells depending upon the specific trigger leading to the development of pain and confirms the need for additional studies on this issue.

## 5. Conclusions

Research on the gut–brain axis has exponentially grown in the last 10 years with more than 5460 papers published compared to about 700 from 1980 to 2014, as indexed in PubMed under the keywords “gut-brain axis”. The role of its dysregulation in different brain pathologies is now clearly emerging, along with the extreme complexity of its intrinsic bidirectional pathways of communication, which include glial cells as fundamental actors. Although at the moment data are limited, it is worth speculating that the gut–glia–neuron connection will progressively prove to be crucially involved in the generation and maintenance of several painful conditions of different origins, including those associated with brain pathologies such as anxiety, depression, and multiple sclerosis. An in-depth understanding of the molecular and biochemical pathways driving this cross-talk is therefore mandatory to its exploitation as an innovative druggable target to approach currently incurable or poorly controlled pain states.

## Data Availability

Data sharing not applicable.
